# Supplementary material for: The Comprehensive Facial Injury (CFI) Score Is an Early Predictor of the Management for Mild, Moderate and Severe Facial Trauma
Source: J Clin Med. 2022 Jun 8;11(12):3281. doi: 10.3390/jcm11123281 (PMC9225200; doi:10.3390/jcm11123281)
Supplement: Supplementary file 1 [file jcm-11-03281-s001.zip › jcm-1624575-supplementary.pdf]

The ROC analysis of Sensitivity and Specificity in detecting those patients at higher risk of surgery procedure is listed in the following table. The cutpoint was choosen to reach the higher compbination of sensitivity and specificity.

Detailed report of Sensitivity and Specificity

Cutpoint	Sensitivity	Specificity	Correctly Classified	LR+	LR-
(>= 1)	100.00%	0.00%	74.68%	1.0000	
(>= 2)	99.81%	20.51%	79.73%	1.2556	0.0093
(>= 2.5)	98.76%	58.99%	88.69%	2.4082	0.0210
(>= 3)	98.67%	58.99%	88.62%	2.4058	0.0226
(>= 4)	88.38%	75.56%	85.14%	3.6165	0.1538
(>= 5)	61.81%	84.83%	67.64%	4.0749	0.4502
(>= 5.5)	44.95%	93.26%	57.18%	6.6679	0.5903
(>= 6)	44.76%	93.26%	57.04%	6.6397	0.5923
(>= 6.5)	35.62%	97.47%	51.28%	14.0893	0.6605
(>= 7)	35.43%	97.47%	51.14%	14.0140	0.6625
(>= 8)	31.33%	98.03%	48.22%	15.9352	0.7004
(>= 9)	24.00%	98.60%	42.89%	17.0880	0.7708
(>= 9.5)	16.19%	99.72%	37.34%	57.6381	0.8405
(>= 10)	16.10%	99.72%	37.27%	57.2990	0.8414
(>= 11)	13.05%	99.72%	34.99%	46.4495	0.8720
(>= 11.5)	11.52%	100.00%	33.93%		0.8848
(>= 12)	11.43%	100.00%	33.85%		0.8857
(>= 12.5)	9.14%	100.00%	32.15%		0.9086
(>= 13)	9.05%	100.00%	32.08%		0.9095
(>= 13.5)	6.95%	100.00%	30.51%		0.9305
(>= 14)	6.86%	100.00%	30.44%		0.9314
(>= 15)	6.38%	100.00%	30.09%		0.9362
(>= 16)	5.62%	100.00%	29.52%		0.9438
(>= 17)	4.67%	100.00%	28.81%		0.9533
(>= 17.5)	4.29%	100.00%	28.52%		0.9571
(>= 18)	4.19%	100.00%	28.45%		0.9581
(>= 19)	4.00%	100.00%	28.31%		0.9600
(>= 20)	3.62%	100.00%	28.02%		0.9638
(>= 21)	3.33%	100.00%	27.81%		1.0000
(>= 22)	0.00%	100.00%	25.32%		0.9667

Obs	ROC Area	Std. Err.	-Asymptotic Normal-- [95% Conf.	Interval]
1406	0.8795	0.0113	0.85742	0.90159

A second ROC analysis of Sensitivity and Specificity was performed to identify patients at higher risk of surgery procedure that took more than 4 hours. The results are listed in the following table.

Detailed report of Sensitivity and Specificity

Cutpoint	Sensitivity	Specificity	Correctly Classified	LR+	LR-
(>= 1)	100.00%	0.00%	11.62%	1.0000	
(>= 2)	100.00%	0.22%	11.81%	1.0022	0.0000
(>= 2.5)	100.00%	1.40%	12.86%	1.0142	0.0000
(>= 3)	100.00%	1.51%	12.95%	1.0153	0.0000
(>= 4)	99.18%	13.04%	23.05%	1.1405	0.0629
(>= 5)	98.36%	43.00%	49.43%	1.7255	0.0381
(>= 5.5)	95.08%	61.64%	65.52%	2.4785	0.0798
(>= 6)	95.08%	61.85%	65.71%	2.4925	0.0795
(>= 6.5)	90.16%	71.55%	73.71%	3.1694	0.1375
(>= 7)	90.16%	71.77%	73.90%	3.1936	0.1371
(>= 8)	88.52%	76.19%	77.62%	3.7172	0.1506
(>= 9)	83.61%	83.84%	83.81%	5.1725	0.1955
(>= 9.5)	76.23%	91.70%	89.90%	9.1871	0.2592
(>= 10)	75.41%	91.70%	89.81%	9.0884	0.2682
(>= 11)	68.03%	94.18%	91.14%	11.6916	0.3394
(>= 11.5)	64.75%	95.47%	91.90%	14.3076	0.3692
(>= 12)	63.93%	95.47%	91.81%	14.1265	0.3778
(>= 12.5)	59.02%	97.41%	92.95%	22.8196	0.4207
(>= 13)	59.02%	97.52%	93.05%	23.8118	0.4203
(>= 13.5)	49.18%	98.60%	92.86%	35.1071	0.5154
(>= 14)	49.18%	98.71%	92.95%	38.0327	0.5149
(>= 15)	45.90%	98.81%	92.67%	38.7242	0.5475
(>= 16)	43.44%	99.35%	92.86%	67.1911	0.5693
(>= 17)	37.70%	99.68%	92.48%	116.6323	0.6250
(>= 17.5)	34.43%	99.68%	92.10%	106.4903	0.6579
(>= 18)	33.61%	99.68%	92.00%	103.9548	0.6661
(>= 19)	31.97%	99.68%	91.81%	98.8839	0.6825
(>= 20)	28.69%	99.68%	91.43%	88.7419	0.7154
(>= 21)	26.23%	99.68%	91.14%	81.1355	0.7401
(>= 22)	24.59%	99.68%	90.95%	76.0645	0.7565
(>= 23)	21.31%	99.68%	90.57%	65.9226	0.7894
(>= 23.5)	18.85%	99.68%	90.29%	58.3161	0.8141
(>= 24)	17.21%	99.68%	90.10%	53.2452	0.8306
(>= 24.5)	16.39%	99.68%	90.00%	50.7097	0.8388
(>= 25)	15.57%	99.68%	89.90%	48.1742	0.8470
(>= 25.5)	12.30%	99.68%	89.52%	38.0323	0.8799
(>= 26)	11.48%	99.68%	89.43%	35.4968	0.8881
(>= 26.5)	9.84%	99.68%	89.24%	30.4258	0.9046
(>= 27)	9.02%	99.68%	89.14%	27.8903	0.9128
(>= 28)	7.38%	99.68%	88.95%	22.8194	0.9292
(>= 29)	6.56%	99.78%	88.95%	30.4265	0.9364
(>= 30)	5.74%	99.78%	88.86%	26.6232	0.9447
(>= 32)	4.92%	99.78%	88.76%	22.8199	0.9529
(>= 35)	3.28%	99.78%	88.57%	15.2133	0.9693

Obs	ROC Area	Std. Err.	-Asymptotic Normal-- [95% Conf.	Interval]
1050	0.9177	0.0141	0.89006	0.94541
